# Mesoporous silica films as hard templates for electrodeposition of nanostructured gold†

**DOI:** 10.1039/d2na00512c

**Published:** 2022-10-10

**Authors:** Tauqir Nasir, Li Shao, Yisong Han, Richard Beanland, Philip N. Bartlett, Andrew L. Hector

**Affiliations:** School of Chemistry, University of Southampton Highfield Southampton SO17 1BJ UK A.L.Hector@soton.ac.uk T.Nasir@soton.ac.uk; Department of Physics, University of Warwick Coventry CV4 7AL UK

## Abstract

Metallic nanostructures have widespread applications in fields including materials science, electronics and catalysis. Mesoporous silica films synthesised by evaporation induced self-assembly and electrochemically assisted self-assembly with pores below 10 nm were used as hard templates for the electrodeposition of Au nanostructures. Electrodeposition conditions were optimised based on pore orientation and size. The growth of nanostructures was initiated at the electrode surface as confirmed by microscopy. The hard templates and Au electrodeposits were characterised electrochemically as well as with X-ray diffraction, small angle scattering and transmission electron microscopy. Finally, mesoporous silica hard templates were removed by hydrofluoric acid etching and stable Au nanoparticles on different electrode surfaces were achieved.

## Abstract 2

Mesoporous silica films synthesised by evaporation induced self-assembly and electrochemically assisted self-assembly, both with pores below 10 nm, were used as hard templates for the electrodeposition of Au nanostructures.

## Graphical abstract

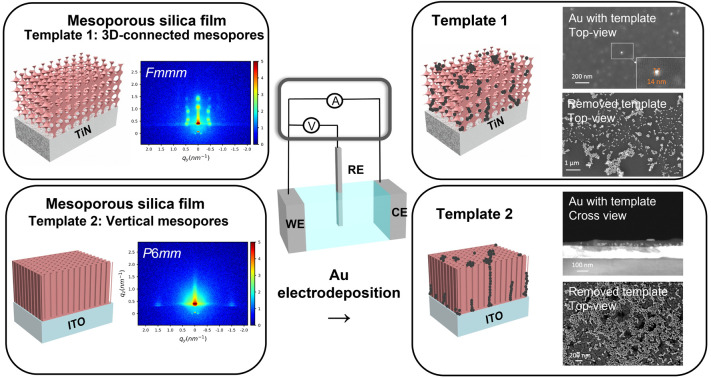

## Introduction

Nanostructures of noble metals with controlled size and distribution are an attractive choice for applications in catalysis, sensing, optics, energy and biomedical devices.^1–5^ Intensive research has been devoted to synthesis and use of gold nanoparticles due to chemical and optical properties which are completely different from bulk gold. In recent years gold nanoparticles (AuNPs) have been widely applied in the field of biology, nanotechnology and particularly catalysis.^6^ The applicability of gold in catalysis is broad, including hydrogen production, fuel cell systems, sensing, pollution control, hydrocarbon combustion, oxidation of carbon monoxide and alcohols.^7–9^ The size of the AuNPs and the support they are deposited on is shown to have a major impact on their performance in catalysis.^8,10^

The synthesis of AuNPs involves top–down or bottom–up approaches. The first is based on breaking down bulk materials into small pieces *e.g.*, by milling and pyrolysis. However, commonly used techniques for nanoparticle synthesis, *e.g.* chemical, electrochemical and synthesis in microbial living systems are based on bottom–up or self-assembly approaches. Chemical reduction is a typical bottom–up method to fabricate gold nanoparticles with the help of reducing agents and stabilising reagents. Zero-to-three-dimensional gold nanostructures with different size and morphologies can be synthesised by chemical reduction. The synthesis of nanostructures by electrodeposition is another old and popular method with certain advantages such as low cost, rapidity, yielding high purity, industrial applications, *etc.*^11^ Hard template assisted electrodeposition is a robust and versatile route to synthesise nanostructures with required size and morphologies. In comparison to chemical reduction approaches in solution, templated electrodeposition could provide gold and other material nanoparticle arrays or networks with better adhesion to a surface and unique morphologies. There is limited reported work for the deposition of nanostructures with the help of templates having pore sizes below 10 nm. The control of deposition in templates in this size range is difficult as is characterisation of the deposits. Efforts have been made to synthesise nanostructures of metals, *i.e.* Au, Ag, Cu, Pt and Pd, by electrodeposition into different kinds of hard templates with pore sizes above 10 nm.^12–18^ Templated electrodeposition provides some control over the size, shape, and distribution of the metal. The template serves two purposes, first it directs a reproducible and organised synthesis and secondly provides the possibility of interconnected nanoparticles.^19^

Anodic aluminium oxide (AAO), track etched membranes and mesoporous silica are often used forms of hard templates for deposition of nanostructures. AAO membranes can be used to produce nanostructures with diameter ranging from 20–400 nm. Track etched membranes are produced through irradiation of a polymer membrane followed by an etching process. Production of nanostructures with sub 10 nm size is difficult with both AAO and track etched membranes.^20,21^ Mesoporous silica films (MSFs) could be used as hard template for smaller size AuNPs deposition but there are only a few literature examples to show this.^14,16,20,22,23^ MSFs are usually produced with sol–gel processes and can be synthesised by evaporation induced self-assembly (EISA), Stöber-solution growth and electrochemically assisted self-assembly (EASA). Stöber-solution growth is based on silicate polymerisation from sol containing silicate, a cationic surfactant and ammonia under specific temperature and requires long time periods (72 h) for formation of very thin mesoporous silica films with vertical pore orientation.^24^ EISA is based on formation of MSFs from a solution containing a silica precursor, a copolymer or cationic surfactant and water–ethanol mixture, with evaporation driving precursor condensation and surfactant organisation.^25^ The films can be formed by dip coating, spin coating or spray coating. The preferred orientation of linear pores in EISA films is usually parallel to the underlying support.^26^ In the EASA process a sol containing water–alcohol mixture, hydrolysed tetra-alkoxysilane and a cationic surfactant can be used to deposit mesoporous silica films on different electrode surfaces by applying a reductive potential.^27^ EASA-derived films possess hexagonal packing of pores having vertical alignment to the electrode surface. Generally, these films are synthesised using cetyltrimethylammonium bromide (CTAB or C_16_TAB) as a surfactant and resulting films contain mesopores having diameter around 2 nm. However, it is challenging to produce nanostructures by electrodeposition in hard templates with 2 nm pore diameter.^17^ Our recent work increased the mesopore size by extending the alkyl chain length of the [Me_3_NR]Br surfactant from 16 to 22 carbons by using quaternary ammonium salts. Mesoporous silica films synthesised with the surfactants C_18_TAB, C_20_TAB and C_22_TAB had larger pore diameters (2.8–4.4 nm) while retaining the ordered structure.^28^

Herein, we report electrodeposition of AuNPs into mesoporous silica film hard templates with pores below 10 nm. The templates have a 3-dimensional pore structure generated by evaporation induced self-assembly with commercial F127 as the surfactant and vertical pores generated by electrochemically assisted self-assembly with C_20_TAB as the surfactant.

## Experimental

Reagents used in this study were tetraethoxysilane (TEOS, 98%, Alfa Aesar), isopropanol (99.5% Fisher), absolute ethanol (99.8%, Fisher), ruthenium hexaamine chloride ([Ru(NH_3_)_6_]Cl_3_, Aldrich), NaNO_3_ (98%, Timstar), KCl (99%, Fisher), KAuCl_4_ (98%, Sigma Aldrich), triblock copolymer F127 (Sigma-Aldrich), eicosyltrimethylammonium bromide (C_20_TAB, synthesised in-house^28^), indium tin oxide (ITO) coated electrodes on glass (surface resistivity 20 Ω sq^−1^. Ossila technologies), HCl (37%, Fisher), hydrofluoric acid (40–45%, Sigma-Aldrich), deionised water (18 MΩ cm, Select Fusion purifier). The titanium nitride (TiN) substrates were made by sputtering (Buehler Helios, rate: 0.135 nm s^−1^) 200 nm of TiN onto a 700 μm thick silicon wafer.

The precursor solution for EISA films was prepared using a method developed by Kataoka and Zhao.^29,30^ Typically a solution was made using 1.0 g TEOS, 5.64 g ethanol, 0.80 g deionised water and 0.10 g 1 mol dm^−3^ hydrochloric acid and was stirred at 338 K for 45 min. Separately, 0.242 g triblock copolymer F127 and 5.64 g ethanol were stirred until F127 was fully dissolved. The above two solutions were mixed and further stirred at room temperature for 60 min. The TiN electrodes were cleaned with acetone then isopropanol for 3 min each by ultrasonication and dried prior to being used for silica film electrodeposition. Dip-coating (dip-coater NIMA IU4 with interface module from Silicon Valley Techparts) was employed for silica film synthesis by the EISA process. The cleaned TiN substrates were vertically immersed into the prepared precursor solution and withdrawn at a rate of 150 mm min^−1^ in 75% relative humidity at 25 °C (Electro-Tech Systems 5506 humidity chamber). The as-made films were aged at 120 °C for 10 h.

For EASA process-based silica films, a sol was made by using 1 : 1 v/v ratio of 0.1 mol dm^−3^ NaNO_3_ and isopropanol followed by addition of 17 mmol dm^−3^ C_20_TAB and 100 mmol dm^−3^ TEOS, pH was adjusted to 3 by adding 0.1 mol dm^−3^ HCl dropwise. The sol was stirred for 2.5 h to achieve maximum hydrolysis. Silica films were electrodeposited onto ITO electrodes with the help of potentiostatic conditions as described elsewhere.^27^ Briefly, electrodes were immersed in the sol and −1.3 V potential *vs.* Ag/Ag^+^ was applied for 20 s. Electrodeposited films were rinsed immediately with deionised water and dried in the oven at 120 °C for 16–24 h.

5 mmol dm^−3^ aqueous [Ru(NH_3_)_6_]Cl_3_ was used for electrochemical characterisation of bare electrodes and of silica films before and after surfactant removal. 0.5 and 1 mmol dm^−3^ KAuCl_4_ dissolved in 0.1 mol dm^−3^ aqueous KCl was used for electrodeposition of AuNPs, the solution was purged under N_2_ before electrodeposition to remove oxygen. Surfactant was extracted from EASA films by soaking in a stirred 0.1 mol dm^−3^ HCl in absolute ethanol solution. For EISA films surfactant was removed by immersion in DCM for 4 h followed by calcination at 350 °C for 5 h. Films without surfactant were used for AuNP electrodeposition by pulsed chronoamperometry. Hydrofluoric acid vapour etching was carried out for 10–20 min on silica films with electrodeposited AuNPs to remove silica by exposing the electrode to vapours above a 48% HF bottle.

All electrochemical experiments were carried out using a three electrode (working, reference and counter) system with a Biologic SP-150 potentiostat. A stainless-steel counter electrode and silver wire pseudo-reference electrode were used for silica film electrodeposition EASA experiments. A Pt mesh counter and Ag/AgCl reference electrode were used in CV characterisation and AuNP electrodeposition experiments. The working electrode in electrodeposition was the ITO or TiN covered with a silica film after surfactant extraction, and a pulsed chronoamperometry regime was applied.

GISAXS patterns were collected using a Rigaku Smartlab with Hypix-3000 detector and Cu K_α_ (*λ* = 1.54 Å) X-rays. The distance between the sample and the detecting surface is around 300 mm. The incident angle was changed according to the critical angle of the samples. In-plane GISAXS patterns were collected by using the detector in 1D mode and full 2D GISAXS images were collected in other cases. High resolution X-ray diffraction patterns were obtained using the same instrument from 10° to 90° 2*θ* with a grazing incidence angle of 1° and the detector in 1D mode. Scanning electron microscopy (SEM) was carried out with a JSM-6500F, a Philips XL30 ESEM and a Zeiss Gemini 500. A Jeol ARM 200F operated at 200 KV was used to collect transmission electron microscopy (TEM) images.

## Results and discussion

### Characterisation of mesoporous silica hard templates

Mesoporous silica hard templates were synthesised onto working electrodes by two processes. The EISA process with Pluronic F127 surfactant produced mesoporous silica films with a 3D arrangement of 8 nm pores diameter (template 1). The templates produced by the EASA method with C_20_TAB surfactant have 4 nm cylindrical pores (template 2). The size and arrangement of the mesopores of the silica films are crucial to electrodeposition of different elements inside these pores as they are the means of connection of the electrolyte to the underlying conducting substrate as well as controlling the locations and dimensions of any deposit. Template 1 was expected to provide easier diffusion and growth due to the larger, 3D interconnected pore structure, whereas template 2 is accessible due to the pore orientation but is likely to be more limiting of diffusion due to the smaller pore size.

**Fig. 1:**
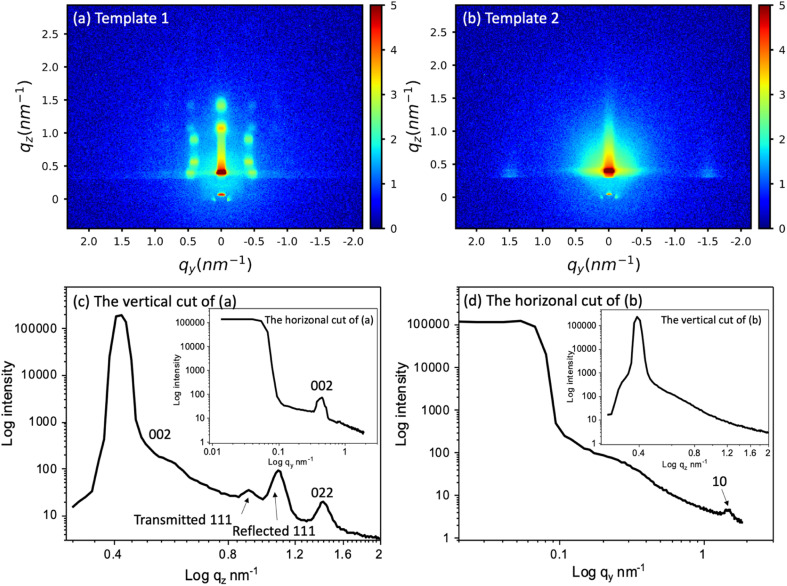
The GISAXS patterns of (a) the mesoporous silica film template 1 synthesised by evaporation-induced self-assembly, (b) template 2 synthesised by electrochemically assisted self-assembly, and (c) and (d) the vertical and horizonal integration of (a) and (b) respectively. The incident angle was 0.3° and scan time set for 20 min. The peak information was calculated using GIXSGUI.^31^

The templates were characterised by GISAXS and microscopy. GISAXS patterns of the two templates were collected to observe the mesostructure, as shown in Fig. 1(a) and (b). Fig. 1(c) and (d) are the vertical and horizonal cuts extracted from (a) and (b) respectively. In the vertical cuts, the peak with highest intensity at around 0.4 nm^−1^ corresponds to the specular reflection (0.428 nm^−1^) and Yoneda peak (0.384 nm^−1^). The two peaks were close together and hence are merged. The multiple diffraction spots of template 1 suggest that a highly ordered mesostructured phase has been synthesised. The GISAXS pattern was compared to simulated GISAXS patterns by using 3D nano-structure indexing in GIXSGUI software. GIXSGUI allows direct indexing of the experimental data within a specific space group as well as labelling the peak positions.^31^ The pattern matches well to the orthorhombic *Fmmm* pore structure with most domains oriented with the [010] axis normal to the substrate. The peaks observed in the vertical cut between *q* = 0.58 nm^−1^ and 1.61 nm^−1^ are the 002, 111 and 022. In the horizonal direction, one peak was observed at 0.451 nm^−1^, which is the 002. The *Fmmm* structure consists of 3D-connected mesopores and could allow deposits to grow along different directions during the electrodeposition process.

The GISAXS of template 2 displays two spots in the equatorial plane, which are identified as the 10 reflections of the 2D-hexagonal *P*6*mm* structure, indicating the vertical orientation of mesopores over a large area of the film. In the horizonal-cut (d), the peak appears at *q* = 1.49 nm^−1^, thus the pore periodicity of the film is *d* = 4.22 nm. The observed faint ring is caused by spherical silica particles incorporated into the surface of the films after silica condensation in the bulk solution during and after the deposition process.^32^ In the vertical direction, no peaks were observed except the specular reflection and Yoneda peak, confirming the vertical pore channels. The vertically oriented pores are expected to allow good electrolyte access to the electrode surface.

**Fig. 2:**
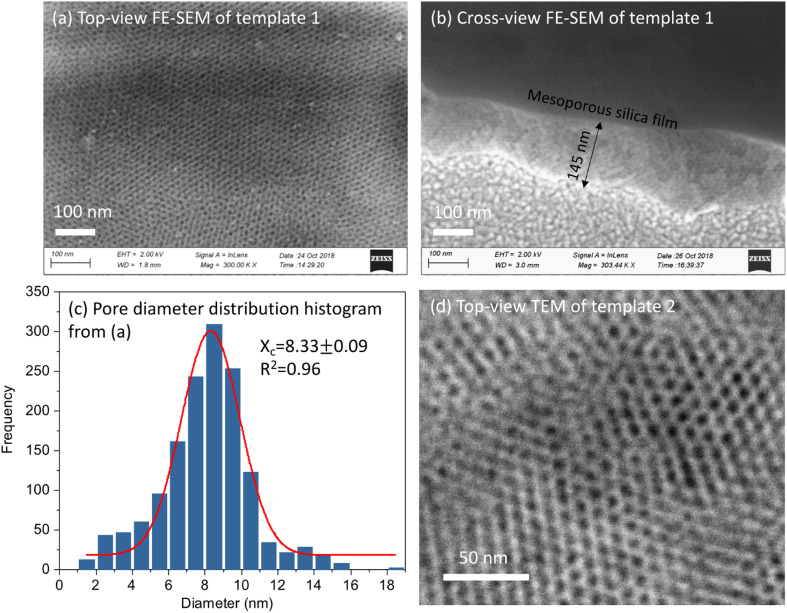
(a) Top-view FE-SEM images of template 1; (b) a cross section FE-SEM image of template 1; (c) the size distribution histogram of the pores in (a); and (d) TEM of template 2. Software “Image J” was used to analyse the pore size distribution and “Origin” was used to draw the histogram. A Gaussian distribution in “Origin” was conducted to fit the histogram and calculate pore size.

Fig. 2(a and b) shows the top-view and cross-view FE-SEM images of template 1. Top-view FE-SEM indicates deposition of a well-ordered mesostructure with high regularity and large domains within the silica film. Pores are hexagonally packed at the surface, corresponding to the *Fmmm* structure obtained from the GISAXS pattern. The histogram shows the size distribution of the pores from (a). The average pore size is 8.33 nm, coefficient of determination *r*^2^ was 0.96. Most of the pore diameters are between 6–10 nm. The cross-view image (b) shows interior pore channels of the film exhibiting well-ordered and crack-free interior mesostructure of silica films with thickness between 120–200 nm. Fig. 2(d) shows a TEM image of EASA films with hexagonal packing of pores which is characteristic of mesoporous films having pores aligned normal to the surface onto which they are deposited. Traditionally, cationic surfactants such as CTAB are used for synthesis of EASA process-based silica films which give rise to a pore size of ∼2 nm.^33^ Herein we used a longer alkyl chain C_20_TAB surfactant and silica films were produced with vertical pore orientation and pore diameter ∼4 nm and film thickness of 100–150 nm.^28^ Nitrogen porosimetry experiments would require a large number of samples to be combined for sufficient material since the mesoporous silica films in this work were approx. 1 × 1.5 cm and 100–200 nm thick. Zhao *et. al.* obtained N_2_ adsorption/desorption curves of mesoporous silica films made by EISA similarly to those used for template 1. The isotherms showed the expected type IV appearance and the pore size was ∼8.5 nm,^30^ in line with our microscopy data. For template 2, in our recent published work, ellipsometric porosimetry with a toluene probe was used to investigate the pore structure of EASA films grown with C_20_TAB.^28^ Again the isotherm had Type IV characteristics and the ∼3.8 nm pore size is in accordance with the size from TEM image herein. The microscopic characterisation of hard templates confirmed the deposition of porous and homogenous silica films.

Electrochemical characterisation by cyclic voltammetry using the redox probe [Ru(NH_3_)_6_]Cl_3_ was employed before and after each step of electrode modification *i.e.*, bare electrode, after silica film formation with surfactant inside the pores and after surfactant extraction (ESI, Fig S1†). Ruthenium hexaamine showed characteristic reversible behaviour on bare electrodes whereas the electrochemical signal was almost completely suppressed with films having surfactant inside the pores, suggesting deposition of homogenous and crack free films. The CV signal was again observed after surfactant extraction which was slightly higher than bare electrodes suggesting complete removal of surfactant and leaving the pores open for mass transport. The slightly higher current after surfactant removal compared with the bare electrode can be explained by the fact that ruthenium hexaamine, being a cationic probe, tends to accumulate inside the mesopores of anionic silica films during CV cycling due to the negatively charged pore walls.^34,35^

### Electrochemical behaviour and deposition of Au into mesoporous silica films

**Fig. 3:**
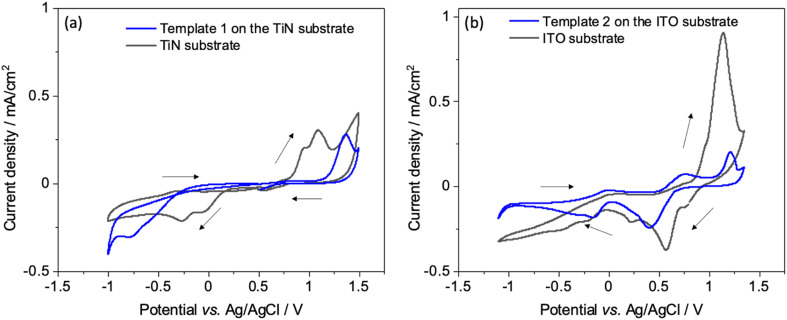
(a) Cyclic voltammograms obtained from an electrolyte of 0.5 mmol dm^−3^ K[AuCl_4_] and 0.1 mol dm^−3^ KCl aqueous solution on a bare TiN substrate (black) and on a mesoporous silica film dip-coated on a TiN substrate (blue). (b) Cyclic voltammograms obtained from an electrolyte containing 1 mmol dm^−3^ K[AuCl_4_] and 0.1 mol dm^−3^ KCl aqueous solution on a bare ITO (black) and on a mesoporous silica film coated on an ITO working electrode (blue). Scan rate 50 mV s^−1^.

Interactions of the [AuCl_4_]^−^ precursor were characterised by CV at unmodified and mesoporous silica modified ITO and TiN electrodes. Fig. 3(a and b) shows the CV with bare TiN and ITO electrodes (black) as well as with the silica film templates (blue). The potential was scanned between 1.4 and −1.1 V as this potential window results in complete reduction of Au^3+^ ions to Au(0).^36^ Two reduction peaks at 0.07 V and −0.27 V on bare TiN substrate and at 0.55 V and 0.25 V on bare ITO substrate were observed, indicating two reduction reactions of [AuCl_4_]^−^. After a wave peak at 0.33 V, diffusion-limited deposition behaviour was displayed on TiN.

When using the mesoporous silica template coated electrodes, the reduction peaks were at more negative potential than the peaks seen with the bare electrodes because of slower diffusion in the mesostructure. Anodic peaks were observed between 0.8 and 1.35 V, they are related to the oxidation (stripping) of the electrodeposited gold species. In the second and third scan for template 1 Fig S2(a),† reduction peaks were shifted to more positive potentials indicating that the gold particles were electrodeposited on gold instead of the electrode surfaces.^37^ There is no obvious change in the reduction peak positions in the case of template 2 Fig S2(b).† The first scan is a characteristic nucleation loop which confirms that greater overpotential is required for nucleation of Au on the electrode surface, and reduction in overpotential for subsequent scans indicates an incomplete stripping process. As a result, gold is deposited more easily on gold than the electrode surface and hence the reduction peaks for these scans are shifted to more positive potentials.^38^ The deposition current with mesoporous silica template covered electrodes was lower than the bare substrates, as expected since the negatively charged [AuCl_4_]^−^ will be repelled by the pore walls. Since the pore walls of mesoporous silica films are negatively charged, diffusion of anionic species through mesopores is restricted due to electrostatic repulsion, as explained by the Donnan exclusion effect.^39,40^ This could also be due to less reactive area of the electrode available due to coverage by insulating silica films. The similar cyclic voltametric behaviour of Au for bare and silica film-coated electrodes demonstrates that most of the mesopores are accessible and thus the silica films are suitable candidates as hard templates for electrodeposition.

Au electrodeposition was performed into mesoporous silica films using a pulsed potentiostatic method with an N_2_ purged aqueous electrolyte containing KAuCl_4_ as precursor. It has been demonstrated that pulsed deposition increases the chances of successful incorporation of metal nanostructures into smaller pore hard templates compared with continuous application of a fixed potential.^17^ Limited diffusion into small pores makes electrodeposition into these structures challenging, so the deposition process has to be precise and controlled.

**Fig. 4:**
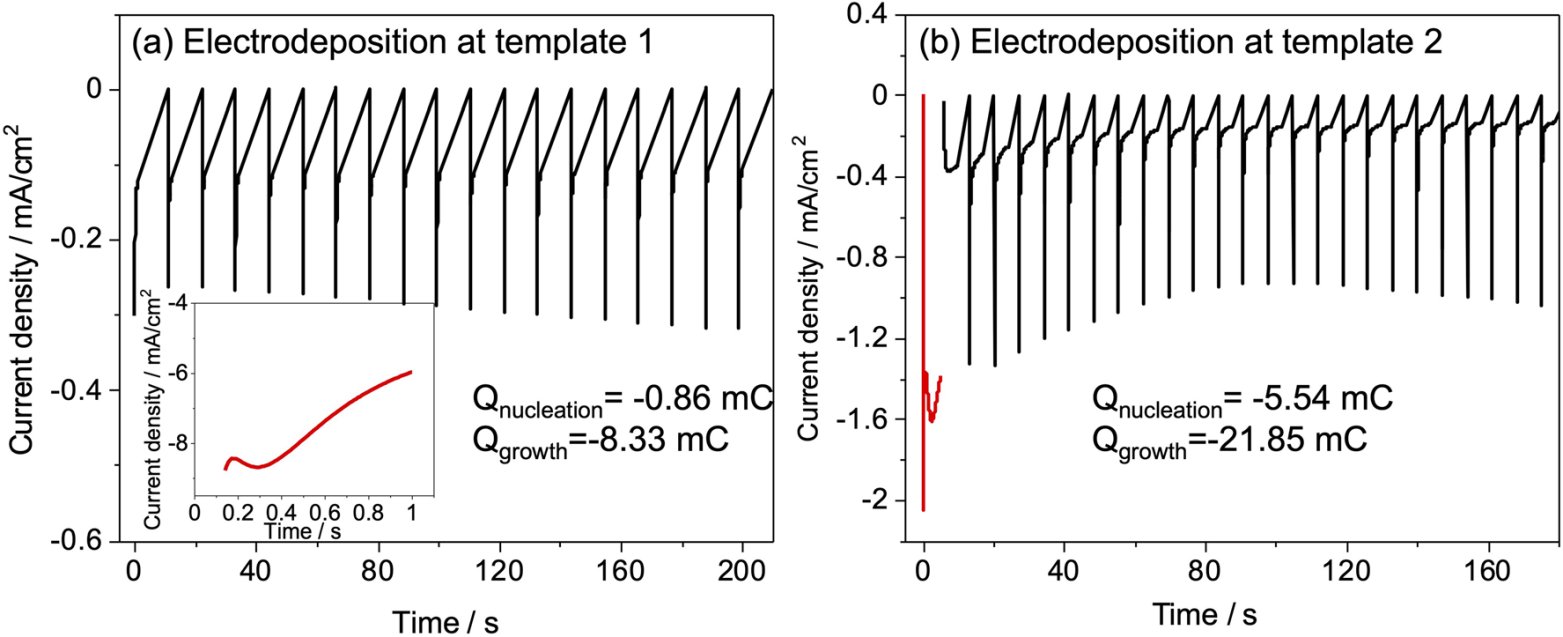
Current transients during pulsed potential deposition (a) in 0.5 mmol dm^−3^ K[AuCl_4_]: nucleation at −1.5 V for 1 s, followed by growth at −0.1 V for 1 s for 100 cycles (template 1), first 20 cycles are shown. (b) In 1.0 mmol dm^−3^ K[AuCl_4_]: nucleation at −1.0 V for 5 s, followed by growth at 0 V for 25 cycles (template 2).

Slow deposition was targeted to avoid the regime in which deposits that nucleate early in the deposition process grow to the surface and then grow more rapidly because diffusion in the bulk electrolyte is faster than in the pore structure. Hence, an electrolyte with a low concentration of K[AuCl_4_] was used and short pulse-on times were applied with pauses in between to allow ion concentrations at the electrode surface to recover. Different values of potential and time for nucleation and pulse on pulse off were tried (Fig. S3†) for both templates and the combination that gave current transients suggesting growth stopping short of the diffusion-limited regime and full recovery before next pulse. Nucleation time was chosen to maximise number of growths at the substrate surface when electrodeposition was carried out with sufficient number of pulses for breakthrough of deposit. Nucleation and period of pulses was optimised based on previous study related to electrodeposition of gold.^37^ Nucleation was performed at −1.5 V for 1 s, followed by growth at −0.1 V for 1 s for 20–100 cycles for template 1. A −1 V potential was applied for 5 s to achieve nucleation followed by deposition at 0 V for a number of pulses ranging from 25–100 for template 2. Fig. 4 shows the current *vs.* time transients for nucleation and pulsed deposition of Au at template 1 and 2.

### Characterisation of Au deposits by diffraction

**Fig. 5:**
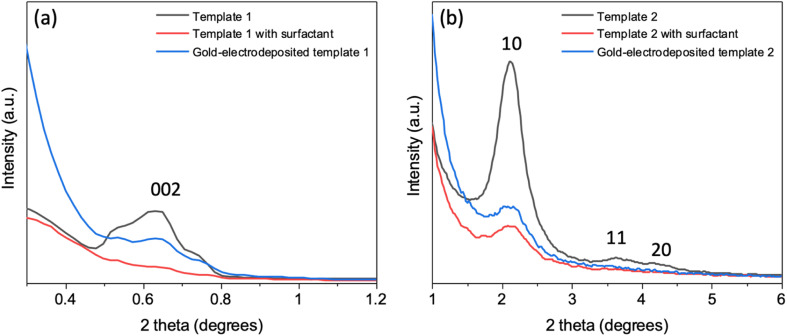
In-plane GISAXS patterns of template 1 (a) and template 2 (b), mesoporous silica film template 1 and 2 (black), template 1 and 2 with surfactant F127 and C_20_TAB (red) and gold-electrodeposited template 1 and 2 (blue). The incident angles for templates 1 and 2 were 0.25° and 0.30°, respectively.

In-plane GISAXS was performed for the silica films with surfactant F127 and C_20_TAB, silica films without surfactants (templates 1 and 2 made with surfactants F127 and C_20_TAB respectively) and silica films after gold was electrodeposited (Fig. 5). For template 1 (Fig. 5a), after surfactant extraction a wide peak was evident at 0.64° (0.45 nm^−1^), which was also observed in the horizontal cut of the GISAXS in Fig. 1. There was no peak observed with surfactant inside the pores due to lack of contrast between the silica and the surfactant. The peak intensity decreased with Au electrodeposition as compared to surfactant extracted film, suggesting that some mesopores were filled with gold. Similar behaviour was observed for peak 1 0 of template 2, which had low intensity with surfactant inside the pores, strong intensity after surfactant removal and weaker intensity after electrodeposition of Au (Fig. 5b). The continuing presence of the in-plane GISAXS features confirmed the presence of long-range order in both templates.

**Fig. 6:**
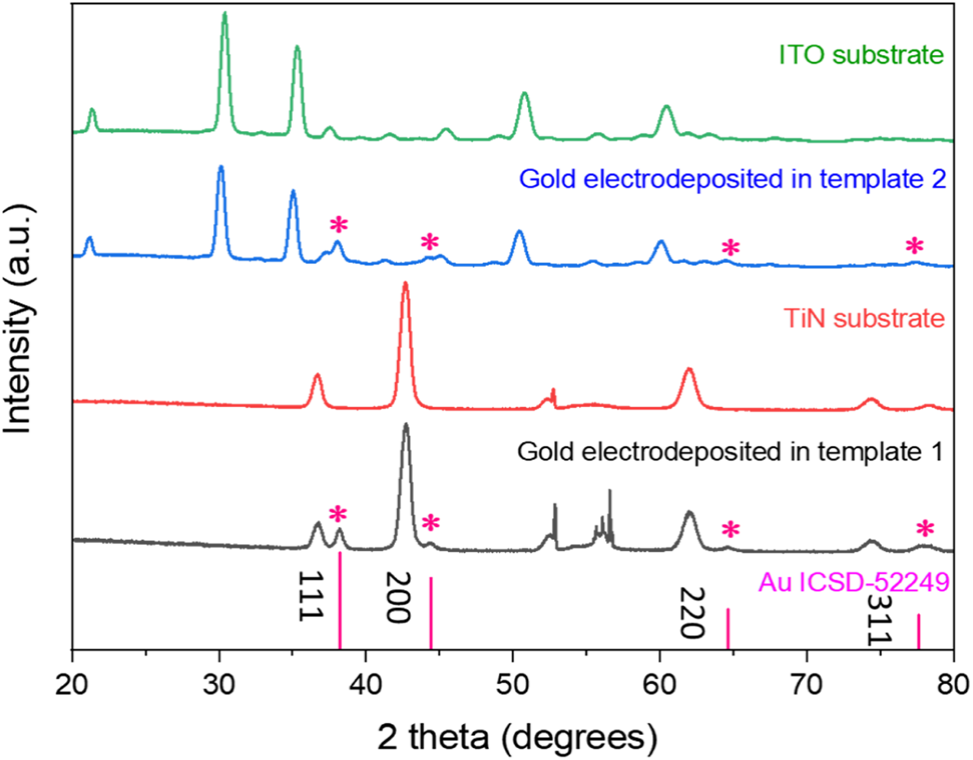
The grazing incidence XRD patterns of gold electrodeposited into templates 1 (TiN substrate) and 2 (ITO substrate), with substrate patterns and the standard intensities for gold shown for comparison.^43^ The incident angle was 1°.

Fig. 6 shows the grazing incidence X-ray diffraction patterns of the mesoporous silica films on the TiN and ITO substrates (templates 1 and 2) and gold electrodeposited into the templates. The peaks arising from the electrodes are in accordance with previously reported peaks for ITO^41^ and TiN.^42^ The Miller indices of the key planes are labelled in the figure. In case of TiN electrodes, the sharp peak at 52.9° comes from the silicon wafer of the substrate. The wide peak between 20° and 30° belongs to the amorphous mesoporous silica. After electrodeposition, FCC-structured gold was observed in both cases in addition to the substrate peaks continuing to be visible.

### Microscopic characterisation of Au deposits

**Fig. 7:**
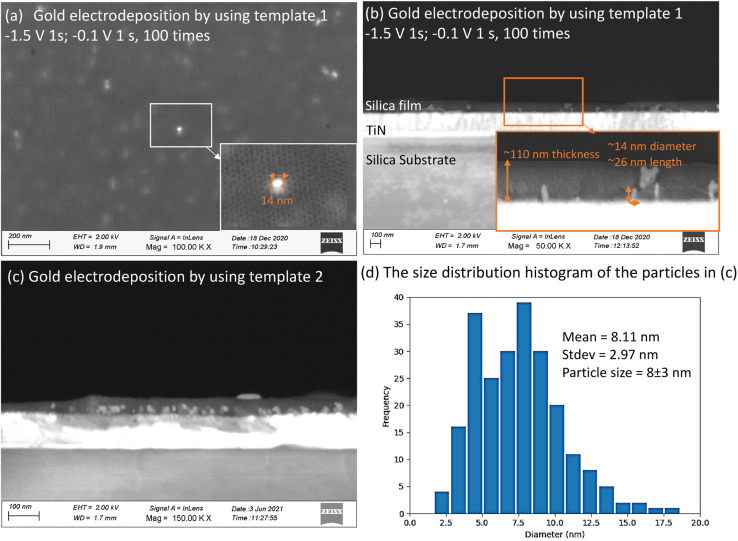
Top-view (a) and cross-view (b) FE-SEM images of gold electrodeposited in template 1; (c) A cross-view FE-SEM image of gold electrodeposited in template 2; (d) the size distribution histogram of the particles in (c). These images were obtained by Zeiss Gemini FE-gun SEM. HF vapour etching of MSFs to reveal Au electrodeposits.

Mesoporous silica films after electrodeposition of Au became pale blue or purple in appearance, the strong absorption band in the visible region due to plasmon resonance in the AuNPs suggesting the incorporation of nanostructured Au.^44^ These films were observed under high resolution SEM (Fig. 7). It can be seen in cross-sectional SEM images that growth starts from the base of MSFs at the electrode surface. These Au structures were nanosized. In the case of EASA films, the average nanoparticle size was measured at around 8 nm, bigger particles were also present, but the majority of these structures were below 10 nm. The size of nanoparticles obtained is in agreement with an already observed phenomenon by Schönenberger *et al.*, where they concluded that metallic nanowires electrodeposited into hard templates had diameter larger by a factor of 3 than the nominal pore diameter of the templates.^21^ The deposited gold particles from template 1 and template 2 are both larger than the mesopores of the templates. It has been previously reported that the AuNPs can grow larger than the size of a mesoporous template.^16,45^ It can be attributed to the pore expansion by the growing gold nanostructures. Also, mesoporous silica films are known to possess some microporosity.^46^ Kanno *et al.* deduced that the Au clusters deposited in micropores may diffuse into mesopores and then crystallize to Au nanoparticles.^22^ Diffusion and crystallisation may result in compressive stress to the pore walls, which could result in formation of Au particles larger than the size of template pores or damage the pore walls and promote growth in the cleavages. It is important to note that average particle size was found to be larger in EISA films (25–100 nm) as the mesopore size is bigger as compared to EASA films and is in accordance with the above stated phenomenon. This suggests a templating role of the MSFs during the growth of nanostructures in directing their morphology and size. TEM images (ESI, Fig. S4†) also confirmed the presence of Au nanoparticles embedded in the base of the silica films. It is worth noting that the growth of the Au started at the electrode surface and particles were observed on the surface of the silica after longer growth times, but nanowires were never found growing all the way through the mesopores by TEM. An explanation for the inability to spot nanowires with the help of TEM in mesoporous silica films was given by Bartlett *et al.*^17^ Once particles grow on the surface, if the metal has any mobility surface energy may be minimised by absorbing the nanowire into the particle which then blocks the pores immediately below it.

**Fig. 8:**
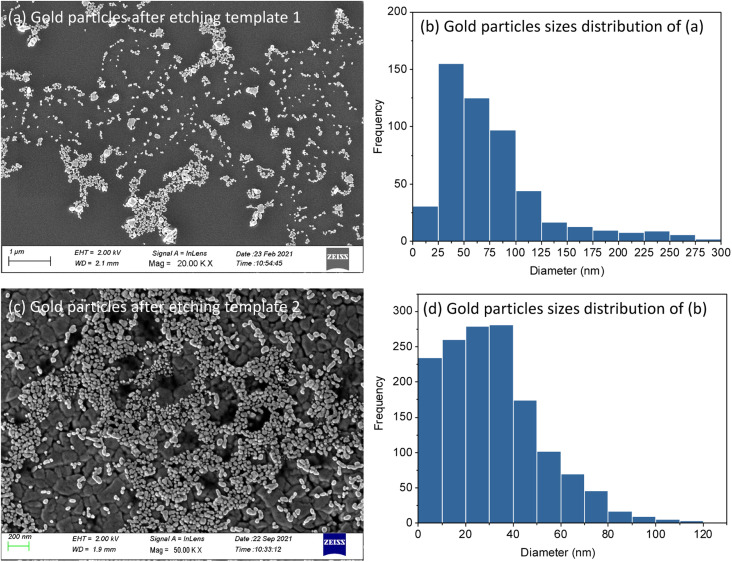
Top-view FE-SEM images of gold nanoparticles arrangements at the surface after etching template 1 (a) and template 2 (c). (b) and (d) are the size distribution histograms of the particles in (a) and (c) respectively.

MSFs with Au electrodeposited were etched by 10–20 min exposure to HF vapour above a bottle of hydrofluoric acid. This process resulted in complete loss of the silica structure (ESI, Fig. S5†) and of the associated GISAXS features (ESI, Fig. S6†). HF-etched samples were further characterised by SEM and XRD. Clusters of nanoparticles were found on the electrode surfaces (Fig. 8). This further confirms that growth of Au nanoparticles starts at the base of MSFs during the electrodeposition process as nanoparticles on the film surface would have fallen away when the silica was etched from underneath them (films were etched facing down toward the open bottle). The mean diameter of the AuNPs calculated from Fig. 8(b) template 1 is 57.1 nm and 22.3 nm from template 2 Fig. 8(d). The template 1 based nanoparticles were larger than that of template 2 which is in accordance with the pore size that controlled the growth and with observations of particles still embedded into the films. The published studies have showed the microporosity of materials templated by PEO-containing triblock copolymers can be tuned to some extent, which provides a route to optimise the mesoporous silica films templates in the future.^47^

## Conclusions

Interconnected nanostructured Au particles were electrodeposited on electrode surfaces through mesoporous silica hard templates with pore size below 10 nm, synthesised by evaporation induced and electrochemically assisted self-assembly processes. The synthesis of Au nanostructures was confirmed by electrochemical, XRD and microscopic characterisation. The size and distribution of nanostructures were found to be dependent on physical properties of hard templates but with structures larger than the template pores. Mesoporous hard templates were etched with the help of hydrofluoric acid leaving behind stable and interconnected Au nanoparticles on electrode surface. The average nanoparticle size indicated that the particle size is related to the pore size of the hard templates. The size of deposited AuNPs is generally larger than the template pore size due to the microporosity and expansion of pores during the Au electrodeposition process. More robust mesoporous templates with minimum microporosity and denser silica pore walls might give rise to more uniform nanostructures.

Smaller sized stable Au nanoparticles have important applications in the field of catalysis, optoelectronics, and sensing. Mesoporous silica films having smaller pore size can be synthesised using surfactants such as Brij S10 and CTAB, even though it is already a significant challenge to deposit into the larger mesopores (∼4 nm and ∼8 nm) in this work. Lowering the microporosity and increasing the density of silica walls in future can enhance the robustness of MSFs to encourage the uniform pore filling and nanostructures growth inside the mesopores instead of growing beyond pore wall.

## Conflicts of interest

The authors declare no conflicts of interest.

## Supplementary Material

NA-004-D2NA00512C-s001
