# Supplementary material for: Mesoporous silica films as hard templates for electrodeposition of nanostructured gold
Source: Nanoscale Adv. 2022 Oct 10;4(22):4798–808. doi: 10.1039/d2na00512c (PMC9642781; doi:10.1039/d2na00512c)
Supplement: NA-004-D2NA00512C-s001 [file NA-004-D2NA00512C-s001.pdf]

Electronic Supplementary Material (ESI) for Nanoscale Advances.
This journal is © The Royal Society of Chemistry 2022

Supplementary Information: Mesoporous silica films as hard templates for electrodeposition of nanostructured gold

Tauqir Nasir[1a], Li Shao[1a], Yisong Han[2], Richard Beanland[2], Philip N. Bartlett[1] and Andrew L. Hector[1]

[1] School of Chemistry, University of Southampton, Highfield, Southampton SO17 1BJ, U.K.

[2] Department of Physics, University of Warwick, Coventry CV4 7AL, U.K.

[a] Authors contributed equally to this work

Email A.L.Hector@soton.ac.uk; T.Nasir@soton.ac.uk

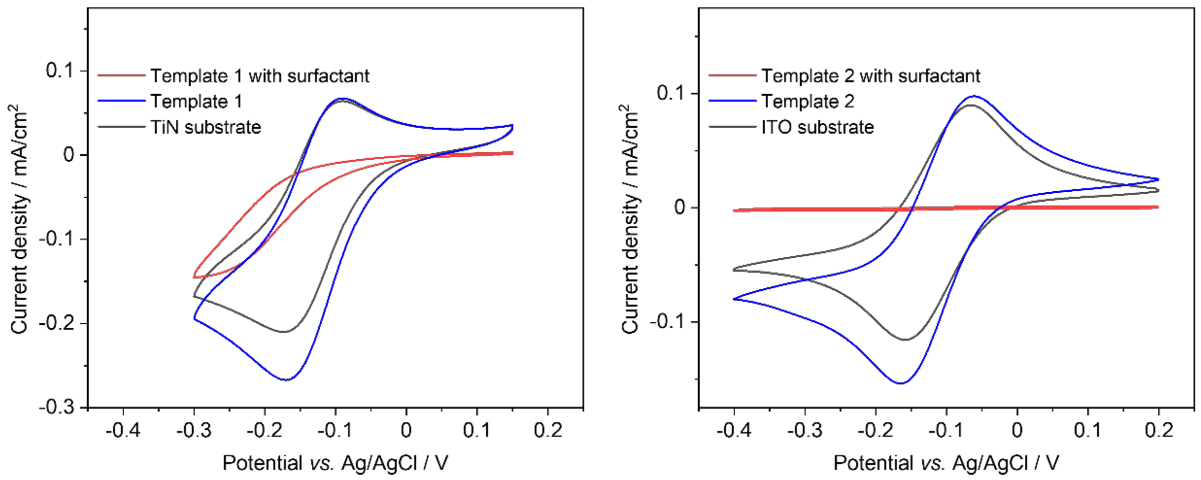

Figure S1: Cyclic voltammograms of 5 mM $[Ru(NH_3)_6Cl_3]$ in 0.1 M $NaNO_3$ (a) at template 1 and (b) template 2 (red) with surfactant inside pores, (blue) open pores without surfactant and (black) bare electrode substrates. Scan rate 50 mv/s

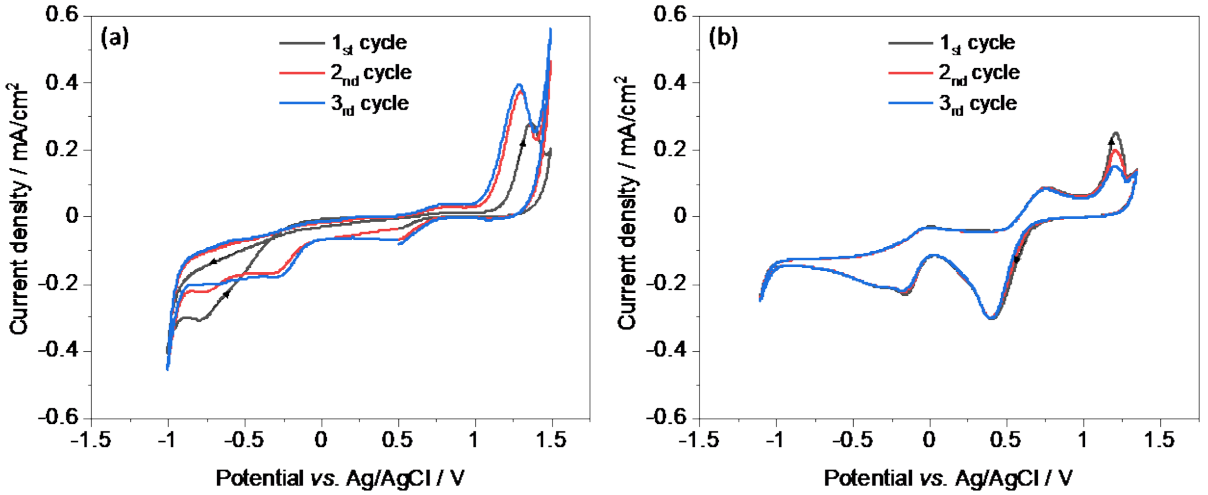

Figure S2: (a) Cyclic voltammograms obtained from an electrolyte of 0.5 mmol dm^{-3} $K[AuCl_4]$ and 0.1 mol dm^{-3} KCl aqueous solution on a mesoporous silica film (template 1) on TiN substrate. (b) Cyclic voltammograms obtained from an electrolyte containing 1 mmol dm^{-3} $K[AuCl_4]$ and 0.1 mol dm^{-3} KCl aqueous solution on a mesoporous silica film (template 2) coated on an ITO working electrode. Scan rate 50 mV/s.

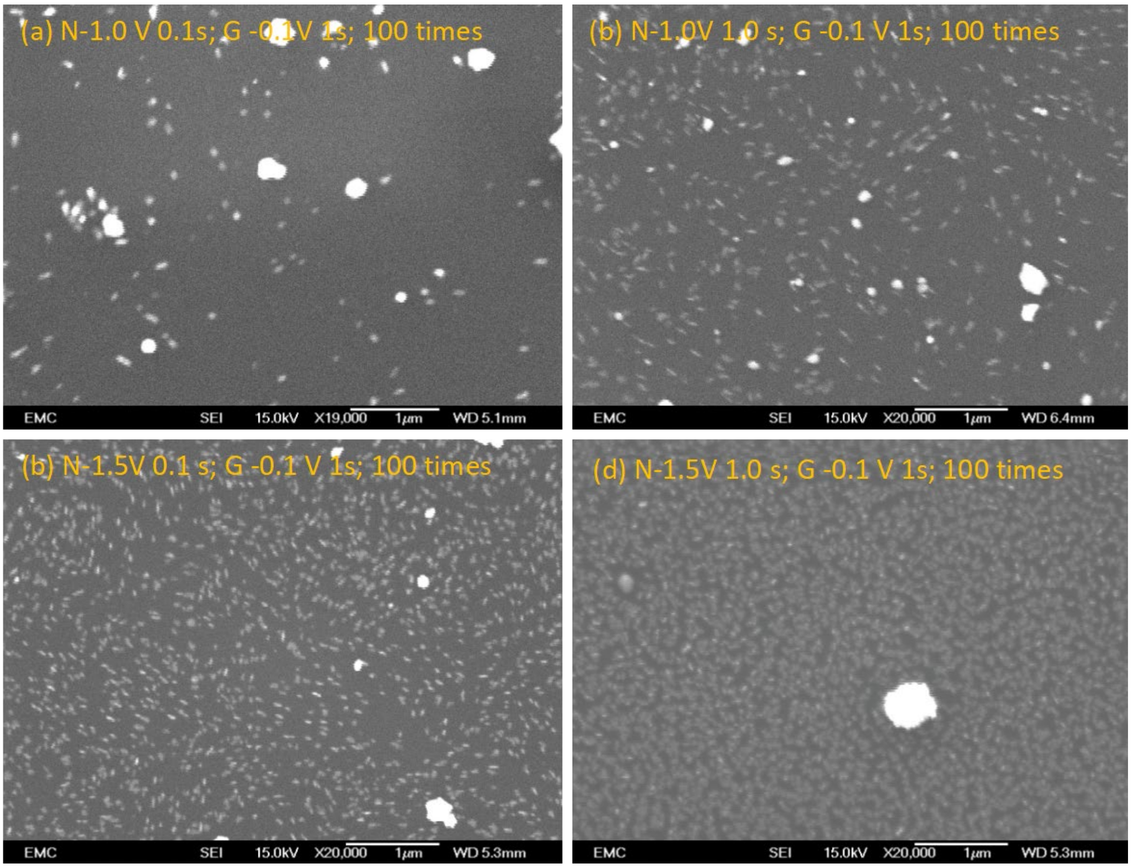

Figure S3: Top-view SEM images of Au electrodeposited in Template 1 under different conditions. Nucleation was at -1.0 or -1.5 V for 0.1 or 1.0 s, as shown, followed in all cases by electrodepositing at -0.1 V for 1 s for 100 cycles.

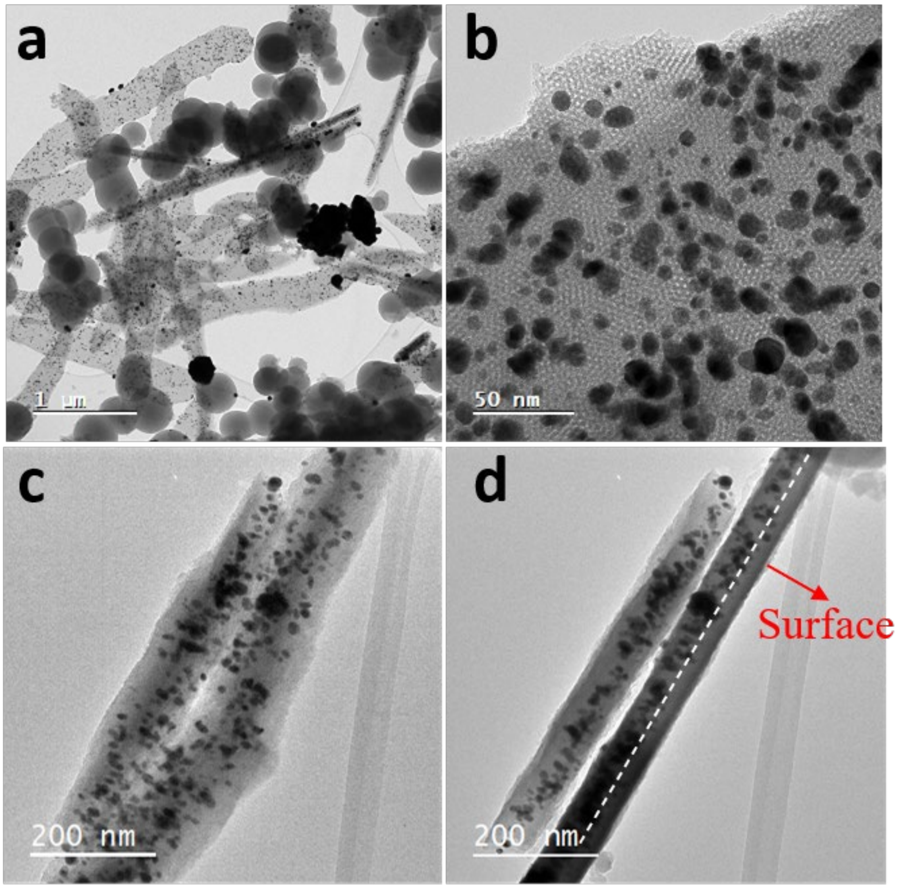

Figure S4: (a, b) Top view TEM images of template 2 after Au electrodeposition. (c, d) The sample is tilted using a high tilt holder to achieve an edge-on condition (looking down the cross section of a piece of the film). The Au particles only appear on bottom side of the silica film.

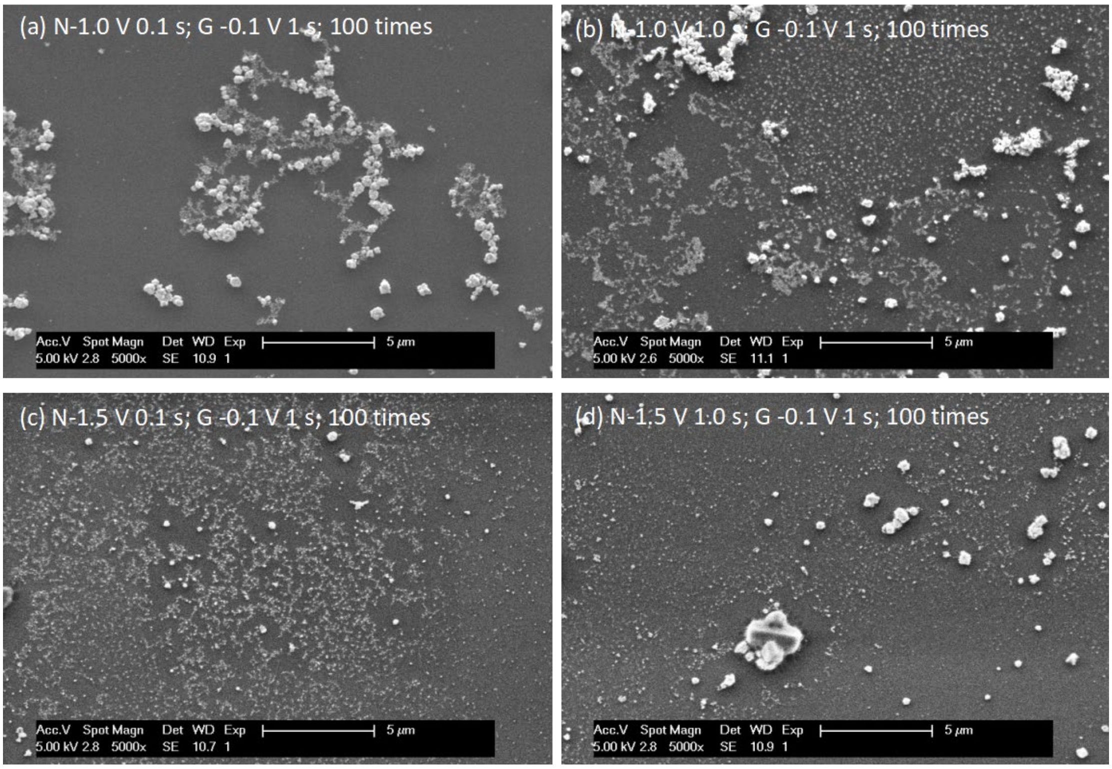

Figure S5: Top-view SEM images after removal of template 1 from Au electrodeposited sample. The electrodeposition conditions were the same as those used in Fig S2.

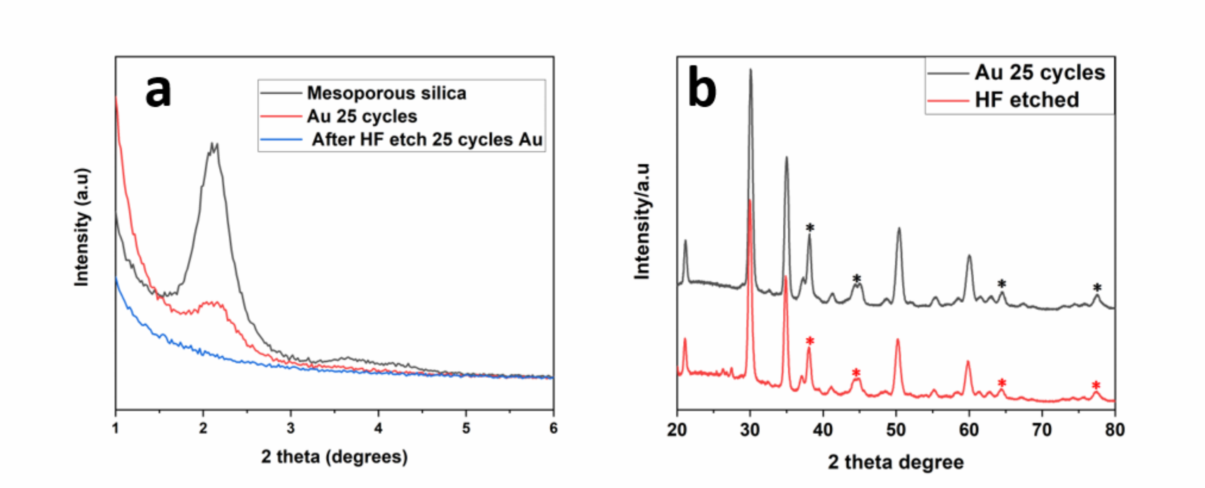

Figure S6: (a) In plane XRD spectra of (black) mesoporous silica, (red) Au electrodeposited into mesoporous silica and (blue) after mesoporous silica removal by HF of Au electrodeposited sample. (b) Wide angle XRD spectra of (black) mesoporous silica and Au (red) after mesoporous silica removal by HF etching of Au electrodeposited sample. (* Au peaks and remainder ITO).
